# Modeling Hourly Productivity of Advanced Practice Clinicians in the Emergency Department

**DOI:** 10.5811/westjem.21298

**Published:** 2025-01-31

**Authors:** Bryan A. Stenson, Peter S. Antkowiak, David T. Chiu, Leon D. Sanchez, Joshua W. Joseph

**Affiliations:** * Beth Israel Deaconess Medical Center, Department of Emergency Medicine, Boston, Massachusetts; † Brigham and Women’s Faulkner Hospital, Department of Emergency Medicine, Boston, Massachusetts; ‡ Brigham and Women’s Hospital, Department of Emergency Medicine, Boston, Massachusetts; ° Contributed equally to this work in the role of co-senior author.

## Abstract

**Introduction:**

Advance practice clinicians (APC) play significant roles in academic and community emergency departments (ED). In attendings and residents, prior research demonstrated that productivity is dynamic and changes throughout a shift in a predictable way. However, this has not been studied in APCs. The primary outcome of this study was to model productivity for APCs in community EDs to determine whether it changes during a shift similar to the way it does for attendings and residents.

**Methods:**

This was a retrospective, observational analysis of 10-hour APC shifts at two suburban hospitals, worked by 14 total individuals. We examined the number of patients seen per hour of the shift by experienced APCs who see all acuity and staff all patients with an attending. We used a generalized estimating equation to construct the model of hour-by-hour productivity change.

**Results:**

We analyzed 862 shifts over one year across two sites, with three shift start times. Site 1 10 am–8 pm saw an average of 13.31 (95% confidence interval [CI] 13.02–13.63) patients per shift; Site 2 8 am–6 pm saw an average of 12.64 (95% CI 12.32–13.06) patients per shift; Site 2 4 pm–2 am saw an average of 12.53 (95% CI 12.04–12.82) patients per shift. Across all sites and shifts, hour 1 saw the highest number of patients. Each subsequent hour was associated with a small, statistically significant decrease over the previous hours. This was most pronounced in the shift’s last two hours.

**Conclusion:**

The productivity of APCs demonstrates a similar pattern of hourly declines observed in both resident and attending physicians. This corroborates prior findings that patients per hour is a dynamic variable, decreasing throughout a shift. This provides further external validity to prior research to include both APCs and community EDs. These departments must take this phenomenon into account, as it has scheduling and operational consequences.

Population Health Research Capsule What do we already know about this issue?

*Patients seen per hour is a key productivity metric. For attendings and residents, it has been shown to be dynamic and changes throughout the course of a shift.*

What was the research question?

*Is this productivity pattern similar for advanced practice clinicians working in community EDs?*

What was the major finding of the study?

*Mean number of new patients seen decreased at each hour of the shift relative to the previous hour (P < 0.01).*

How does this improve population health?

*Understanding how many patients are seen at each hour of the day, based on clinician type and hour of shift, could inform staffing models and help throughput.*

## INTRODUCTION

Advanced practice clinicians (APC) play a significant role in the care provided in many academic and community emergency departments (ED). APCs are non-physician clinicians, such as nurse practitioners (NP) and physician assistants (PA), who see and evaluate patients under the supervision of, and in collaboration with, attending physicians. The use of APCs has increased in the past few decades, with the most recent National Hospital Ambulatory Medical Care Survey in 2020 estimating that 10.1% of ED visits involved an NP, and 13.4% of visits involved a PA.^1^

Previous literature on APCs in the ED is limited but has ranged from analyzing resource utilization to describing overall trends in how APCs are used and in which practice settings.^2^ ^,^ ^3^ One group found that APCs saw more patients per hour and generated more relative value units (RVU) per hour—both key markers of productivity—than a resident physician in a fast track setting, while generating fewer RVUs per patient.^4^ This trend held up in a higher acuity setting in this same group.^5^ However, with such a significant portion of ED visits involving an APC, there is still limited data on overall productivity.^6^

Among the metrics commonly used to measure clinical productivity, patients seen per hour is one of the most essential to ED operations planning. It often leads to important staffing decisions at all types of EDs, ranging from large academic EDs to small community ones.^7^ While productivity is often thought of as a static quantity measured across a shift, in reality it is dynamic and changes throughout the course of a shift in a predictable way. This phenomenon has been demonstrated in emergency medicine (EM) attendings and residents, and it manifests as a stepwise decrease in productivity after the first few hours of a shift.^8^ ^,^ ^9^ This behavior was similar in both of these groups and is consistent with the lived experience of working in an ED. When a physician shows up fresh to a shift, they have more bandwidth to see new patients. After a few hours, as each of those patients starts to have results return and require additional decisions, there is less time to see new patients.

Accounting for this phenomenon can have significant operational impacts. By understanding the true hourly capacity of the workforce, administrative leadership can ensure this best matches up with the hourly patient demand.^10^ This has the potential to improve key operational metrics such as door-to-clinician time and the rate of patients that leave without being seen, a metric that is itself not static and is impacted by various departmental factors.^11^ To date, however, this pattern has not been studied in APCs practicing in the ED. Our primary outcome in this study was to determine whether this pattern was similar for APCs working in a community ED, as this would provide further external validation of the previous model to the community setting and to a relatively new group of the workforce.

## METHODS

We performed a retrospective, observational analysis of APC shifts at two suburban hospitals in the Northeastern United States from July 1, 2020–June 30, 2021. Site 1 saw an average daily volume of 54.46 patients with an Emergency Severity Index (ESI) score mix of 1.48% ESI 1; 30.00% ESI 2; 50.65% ESI 3; 17.28% ESI 4, and 0.58% ESI 5. Site 2 had an average daily volume of 79.71 patients with an ESI score mix of 1.01% ESI 1; 26.7% ESI 2; 49.6% ESI 3; 20.4% ESI 4; and 2.3% ESI 5.

For the attending shift schedule, site 1 had a shift schedule of 7 am–4 pm, 2 pm–11 pm, and 10 pm–7 am for the first six months of the study. For the second six months the schedule changed to 7 am–2 pm, 12 pm–7 pm, 4 pm–11 pm, and 11 pm–7 am to add more attending coverage. At site 2, the shifts were 7 am–3 pm, 12 pm–9 pm, 3 pm–11 pm, and 10 pm–7 am. The sign-out culture at both sites is that patients will have an established plan for disposition prior to transitioning to the new team.

At both sites, APC shifts are 10 hours long. At the first site, there is a single daily APC shift from 10 am–8 pm. At the second site, there were two APC shifts during the study period, from 8 am–6 pm and from 4 pm–2 am. There were several days during which the first site had no APC coverage, and the second site had only a single shift. Five APCs worked the shifts at site 1, including a mix of both NPs and PAs, while 10 APCs worked the shifts at site 2, consisting solely of PAs. One of the PAs worked shifts at both sites during the study period. In total there were 14 APCs, 2 NPs and 12 PAs. The APCs saw all levels of patient acuity. These sites employ a shared-visit model, and all patients seen by an APC are presented to, and then evaluated by, an attending physician. The APCs continue to pick up new patients throughout the shift and are not limited in doing so by attending availability to staff. Over 70% of the APCs in the study had >5 years of clinical experience at the start of the study period.

We used a de-identified quality assurance database for this study, which is primarily used for operations planning. The database is automatically populated by the sites’ electronic health record (EHR). Timestamps of patient arrivals, APC assignments, and patient dispositions are automatically recorded by the EHR. The timestamp data is compiled along with additional aggregated and de-identified patient-level data, in accordance with HIPAA-SAFE HARBOR criteria, prior to data analysis. Only the patients seen by an APC were included for analysis, and registration anomalies had already been removed. This study was granted an exemption of informed consent, as part of a larger project usiing a de-identified administrative dataset of ED throughput for quality assurance purposes. The exemption was granted by the institutional review board affiliated with the clinical sites, which includes direct involvement by patient and community representatives in the oversight and approval of all research protocols.

The primary outcome was the number of new patients seen at each hour of a standard 10-hour APC shift. We used a generalized estimating equation to construct the model of APC productivity, with the individual shift as the grouping in light of the use of multiple hourly measurements from the same shift. A Poisson distribution with a log link was used, as the outcome variable (patients seen in an hour) reflects a positive count variable in a fixed time interval. We evaluated the model using an autoregressive covariance structure, with alternate covariance structures tested in sensitivity analyses. The hour of the shift and the shift time and location were used as covariates. We report final parsimonious models as determined by quasi-likelihood score. A two-sided *P*-value <.05 was considered statistically significant, with strict correction for multiple comparisons. For the purposes of model interpretability, we report the calculated model predictions, with the raw (exponential) model covariates in a supplemental appendix. Analysis was conducted in Python 3.11 using the Statsmodels and SciPy packages (Python Software Foundation, Wilmington, DE).

## RESULTS

During the study period (July 1, 2020–June 30, 2021), we analyzed 862 shifts, of which 345 were at Site 1 (single coverage), and 517 were at Site 2 (two-shift coverage). All the worked shifts in this period were included in the study, and no aberrant timestamps were found in the database, meaning that the timestamp of APC assignment always aligned with the shift hours on the schedule. Not every APC shift was staffed during the study period, due to factors including quarantine, operational reassignments within the network, and staffing shortages from the COVID-19 pandemic.

Site characteristics are summarized in Table 1. At Site 1 with a single APC shift from 10 am–8 pm, APCs saw a mean of 13.31 patients per shift (95% CI 13.02–13.63). At Site 2, APCs saw 12.64 (95% CI 12.32–13.06) patients during the 8 am–6 pm shift, and 12.53 (95% CI 12.04–12.82) patients during the 4 pm–2 am shift. While small, these differences were statistically significant (*P* < 0.01 for all pairwise comparisons).

**Table 1.** Characteristics of the study participants and sites evaluated.

Characteristic	Site 1	Site 2
Approximate yearly visits	23,000	33,000
Shifts per day	1	2
Shifts evaluated	345	517
APCs working during the study period	5	10
Mean patients per shift	13.31 (95% CI 13.02–13.63) [10 am–8 pm]	12.64 (95% CI 12.32–13.06) [8 am–6 pm] 12.53 (95% CI 12.04–12.82) [4 pm–2 am]

*APCs*, advance practice clinicians.

Across all sites and shifts, the first hour of the shift demonstrated the highest number of patients seen (Site 1: 2.25 [95% CI 2.17–2.33], Site 2 8 am–6 pm: 2.12 [95% CI 1.98–2.26], and Site 2 4 pm–2 am: 2.10 [95% CI 1.95–2.26]). Each hour was associated with a small, but statistically significant decrease over the previous hours (Table 2). This decrease was most pronounced during the last two hours of the shift, leading to an average well below a single patient seen per hour during hours 9 (Site 1: 0.57 [95% CI 0.50–0.64], Site 2 8 am–6 pm: 0.54 [95% CI 0.46–0.62], Site 2 4 pm–2 am: 0.53 [95% CI 0.45–0.62]) and 10 (Site 1: 0.14 [95% CI 0.11–0.17], Site 2 8 am–6 pm: 0.13 [95% CI 0.10–0.17], Site 2 4 pm–2 am: 0.13 [95% CI 0.10–0.17]). This trend can be visualized in Figure. A sensitivity analysis did not reveal any significant difference in hourly volume of patients seen by APCs by day of week. Prior research at these hospitals has shown adequate hourly patient volumes suggesting there is not a shortage of patients to be seen.^9^

**Table 2.** Models of new patients seen per hour.

Site 1: 10 am–8 pm shift		
Shift hour	Mean new patients (95% CI)	*P*-value
1	2.25 (2.17–2.33)	< 0.01
2	1.96 (1.80–2.13)	< 0.01
3	1.80 (1.65–1.96)	< 0.01
4	1.66 (1.52–1.81)	< 0.01
5	1.42 (1.29–1.50)	< 0.01
6	1.33 (1.21–1.46)	< 0.01
7	1.26 (1.14–1.39)	< 0.01
8	0.98 (0.89–1.09)	< 0.01
9	0.57 (0.50–0.64)	< 0.01
10	0.14 (0.11–0.17)	< 0.01
**Site 2: 8 am–6 pm shift**		
**Shift hour**	**Mean new patients (95% CI)**	***P*-value**
1	2.12 (1.98–2.26)	< 0.01
2	1.85 (1.65–2.07)	< 0.01
3	1.69 (1.5–1.91)	< 0.01
4	1.56 (1.38–1.76)	< 0.01
5	1.33 (1.18–1.50)	< 0.01
6	1.25 (1.10–1.42)	< 0.01
7	1.19 (1.04–1.35)	< 0.01
8	0.92 (0.81–1.06)	< 0.01
9	0.54 (0.46–0.62)	< 0.01
10	0.13 (0.10–0.17)	< 0.01
**Site 2: 4 pm–2 am shift**		
**Shift hour**	**Mean new patients (95% CI)**	***P*-value**
1	2.10 (1.95–2.26)	< 0.01
2	1.83 (1.63–2.06)	< 0.01
3	1.68 (1.48–1.90)	< 0.01
4	1.55 (1.37–1.75)	< 0.01
5	1.32 (1.16–1.50)	< 0.01
6	1.24 (1.09–1.41)	< 0.01
7	1.18 (1.03–1.35)	< 0.01
8	0.92 (0.80–1.05)	< 0.01
9	0.53 (0.45–0.62)	< 0.01
10	0.13 (0.10–0.17)	< 0.01

*CI*, confidence interval.

**Figure.**
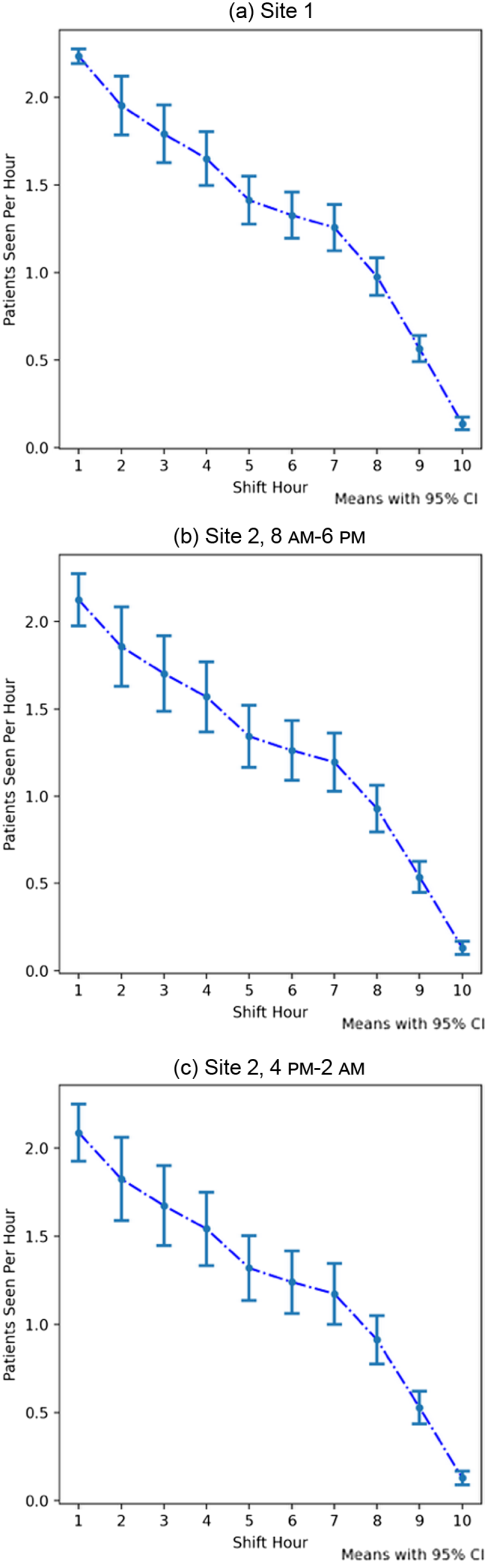
Mean number of patients seen per shift hour by advanced practice clinicians. *APC*, advanced practice clinician.

## DISCUSSION

Our findings in this study suggest that APCs may demonstrate a similar pattern of hourly declines in productivity that has been observed in both resident and attending physicians.^8^ ^,^ ^9^ This corroborates prior findings that suggest that patients seen per hour is a dynamic variable. An intuitive explanation of this finding follows from the fact that patient evaluations take place over multiple hours of a shift, and that seeing a new patient later in the shift requires the APC to balance the demands of seeing an additional patient with concurrently caring for existing patients. The APCs may see more patients earlier in the shift precisely because they have the greatest cognitive bandwidth at the start of a shift, with no active patients. As those patients start to generate results and require re-evaluation, interpretation of imaging or labs, or procedures that add to the cognitive load for an APC, they will see fewer new primary patients.

However, there are substantial differences in the patterns we have observed in APCs relative to patterns of physician productivity previously described in the emergency medicine operations literature.^8^ ^,^ ^9^ Notably, while all of these groups demonstrate progressive declines in hourly productivity and see a higher proportion of patients in the first few hours of their shifts, the APCs in our study demonstrated both a smaller “peak” at the beginning of their shifts compared to those reported with attending physicians, and a more gradual decrease from hour-to-hour relative to resident physicians. The cause of this is likely multifactorial; however, in the prior studies for both attendings and residents, those groups were incentivized and graded on productivity; the APCs in our study did not have the same explicit tie to productivity.

This has important downstream consequences when creating staffing models. While shifts typically span 10 hours and there is an administrative expectation for equal capacity during all hours of coverage, the 9^th^ and 10^th^ hours of a shift do not provide much in the way of new patient evaluations. So, when hiring and staffing a department and trying to best align the number of hourly arrivals with the available staff (residents, attendings or APCs) the administration must take this pattern into account. Understanding how many patients are expected to be seen at a specific hour of the day, based on what staff are available and the hour of each person’s shift, may help throughput.

## LIMITATIONS

Our study does have many limitations. It was only done at two community hospitals in a similar geographic region. Because there were only three shift start times, there was less variability than prior studies performed on resident and attending physicians, which also had a greater variety of shift starting and ending times, including overnights. However, as long as there were adequate patients to be seen at each hour of the day—as seen in prior studies of attending independent productivity at these sites—this limitation should be mitigated. There were also two hours of overlap between shifts at the second site, which may have contributed to some productivity drop-off for the 8 AM–6 PM shift at site 2. Additionally, within this network APCs cared for all levels of patient acuity, and each visit required staffing and evaluation by an attending physician. This differs from other models where APCs can discharge lower acuity patients without an attending evaluation.

While the delay of waiting for an attending to see the patient may prolong some tasks and decisions, this group of APCs had a lot of experience and independence (>70% with over five years of experience) and continued to pick up new patients in the interim. Further, at the two study sites APCs were used to see patients primarily, and this may not be applicable to other ways they are used in departments, such as managing observation patients. Lastly, as this study was conducted at two small community sites there were only a few total APCs (14 total individuals) who primarily work only at a single site, and this group may not be representative of larger groups of APCs or those working in multiple hospital or urgent care settings.

## CONCLUSION

Our findings suggest that the productivity of advanced practice clinicians may follow a pattern of decreasing over successive hours of a shift, similar to both attendings and residents. This study reinforces prior literature that demonstrates that patients per hour is a dynamic variable, which starts at its highest point and decreases significantly each subsequent hour. By verifying that this pattern is consistent in APCs, it broadens the productivity model of prior research. Community EDs, which are often staffed with APCs and have no resident coverage, may need to take this phenomenon into account as it has significant scheduling and operational consequences.

## Supplementary Information




