# Supplementary material for: Modeling Hourly Productivity of Advanced Practice Clinicians in the Emergency Department
Source: West J Emerg Med. 2025 Jan 31;26(2):295–300. doi: 10.5811/westjem.21298 (PMC11931699; doi:10.5811/westjem.21298)
Supplement: Supplementary file 1 [file wjem-26-295-s001.docx]

Supplemental Table 1 – Raw Model Estimation of New Patients Seen per Hour

| Variable | Coefficient | 95% CI | P-Value |
| --- | --- | --- | --- |
| Intercept (Hour 1, Shift: 10AM) | 0.8098 | 0.774 – 0.846 | < 0.001 |
| Hour 2 | -0.1360 | -0.183 – -0.089 | < 0.001 |
| Hour 3 | -0.2242 | -0.277 – -0.172 | < 0.001 |
| Hour 4 | -0.3056 | -0.358 – -0.253 | < 0.001 |
| Hour 5 | -0.4636 | -0.519 – -0.408 | < 0.001 |
| Hour 6 | -0.5266 | -0.586 – -0.467 | < 0.001 |
| Hour 7 | -0.5794 | -0.643 – -0.516 | < 0.001 |
| Hour 8 | -0.8289 | -0.896 – -0.762 | < 0.001 |
| Hour 9 | -1.3744 | -1.461 – -1.287 | < 0.001 |
| Hour 10 | -2.7818 | -2.969 – -2.595 | < 0.001 |
| Shift: 8AM (Site 2) | -0.0688 | -0.105 – -0.032 | < 0.001 |
| Shift: 4PM (Site 2) | -0.0604 | -0.092 – -0.029 | < 0.001 |

Model predictions use coefficients with an exponential link function (e.g. mean patients seen in Hour 1 of the 10AM shift = e^(0.8098) = 2.475)
